# Ebselen: A promising therapy protecting cardiomyocytes from excess iron in iron-overloaded thalassemia patients

**DOI:** 10.1515/med-2023-0733

**Published:** 2023-07-12

**Authors:** Mobin Ghazaiean, Aily Aliasgharian, Hossein Karami, Hadi Darvishi-Khezri

**Affiliations:** Student Research Committee, Mazandaran University of Medical Sciences, Sari, Iran; Thalassemia Research Center (TRC), Hemoglobinopathy Institute, Mazandaran University of Medical Sciences, Sari, Iran; Department of Pediatric, School of Medicine, Thalassemia Research Center (TRC), Hemoglobinopathy Institute, Mazandaran University of Medical Sciences, Sari, Iran

**Keywords:** iron-overloaded, β-thalassemia, cardiomyopathy

## Abstract

Iron-overload-associated cardiomyopathy has been one of the primary causes of mortality in thalassemia patients with iron burden. There is growing evidence citing the beneficial effects of ebselen as an antioxidant selectively blocking the divalent metal transporter 1 (DMT-1) to deter iron ingress into cardiomyocytes, raising internets in viewing this component in this population in order to treat and even prevent cardiomyopathy occurring from iron surplus. In this article, we reviewed the potential advantageous effects of ebselen in thalassemia patients who suffer from iron excess, susceptible to cardiomyopathy induced by iron overload. A systematic search in several databases, including PubMed, Scopus, and Web of Science, was conducted to explore the role of ebselen in controlling iron-overload-related cardiomyopathy in thalassemia patients by the keywords of Ebselen AND iron. The inclusion criteria were English-written preclinical and clinical studies investigating the efficacy and side effects of ebselen in an iron-overload context. After searching the databases, 44 articles were found. Next, of 19 published articles, 3 were included in this article. After reviewing the references of the included studies, no articles were added. In conclusion ebselen can be a promising adjuvant therapy in patients with thalassemia alongside the standard treatment with iron chelators, particularly in severe cases with cardiomyopathy, due to falling iron inflow by inhibiting DMT-1 and increasing ferroportin-1 expression and antioxidant properties. However, clinical studies need to be carried out to reach a definite conclusion.

## 1 Introduction

In patients with hemoglobinopathies, especially β-thalassemia cases, excess iron deposition, typically induced by transfusion strategies, causes secondary iron overload. Iron burden in these patients results from inefficient erythropoiesis and hyperabsorption of iron from the intestine. Excess iron accumulation in these patients is often associated with red blood cell transfusion, damaging vital organs. Excess iron subsided in target organs, such as the heart, liver, and pancreas, has been a challenging problem in these cases [1]. Despite frequent blood transfusions in these patients, it has been reported that the time of hemosiderosis development varies organ by organ, which takes place in the heart as a late, patchy, dispensed form of deposition [2].

Thalassemia is one of the most common monogenic disorders of inherited hemoglobinopathies requiring a multidisciplinary approach to medical management due to varied genotypes and phenotypes. Heart failure is one of the serious ramifications that have ramped up the morbidity and mortality in these patients. The major issue is iron subsides in the cardiomyocytes, thereby leading to problems in the electrophysiological and mechanical functions in the heart, i.e., dysrhythmia and heart failure caused by myocardial dysfunction [3]. As a sequel, iron accumulation alongside damaged cell membranes and ion channels by escalated oxidative stress in cardiomyocytes impinge adversely on cardiac electrical activity, consequently disrupting the heterogeneous conduction and developing re-entry phenomenon. Precisely, smaller overshoot potential derived from a dropped rapid depolarization following decreased Na+ currents puts cardiomyocytes at risk for dysrhythmia, which accompanies shorter action potential duration caused by a reduction in the late fast sodium current during the plateau phase. Intra-atrial tachycardia, atrial fibrillation, and flutter are the most prevalent dysrhythmia in β-thalassemia, while malignant ventricular arrhythmias are more seen in severe excess iron burden [4].

There are several new pharmacological approaches that are intriguing in conjunction with iron chelators to alleviate cardiac hemosiderosis more effectively, including antioxidants and mitochondrial dynamics modulators [5]. Ebselen, as an imitator glutathione peroxidase (GPx), has provided some evidence for improving cardiac function in iron-overloaded conditions. In this article, we reviewed the potential advantageous effects of ebselen in thalassemia patients who suffer from iron excess, susceptible to cardiomyopathy induced by iron overload.

## 2 Methods

To look at the role of ebselen in controlling iron-overload-related cardiomyopathy in thalassemia patients, a systematic search in several databases, including PubMed, Scopus, and Web of Science, was conducted using the keywords: Ebselen AND iron. The inclusion criteria were English-written preclinical and clinical studies investigating the efficacy and side effects of ebselen in an iron-overload context. Emphasis has been placed on studies evaluating anti-inflammatory and antioxidant properties of ebselen. The investigations were initially checked. Then, the researchers independently assessed the full text of the final included studies. The references of the included studies were reviewed not to miss any relevant articles. At the next step, the details of the included studies were extracted.

## 3 Results

**Figure 1:**
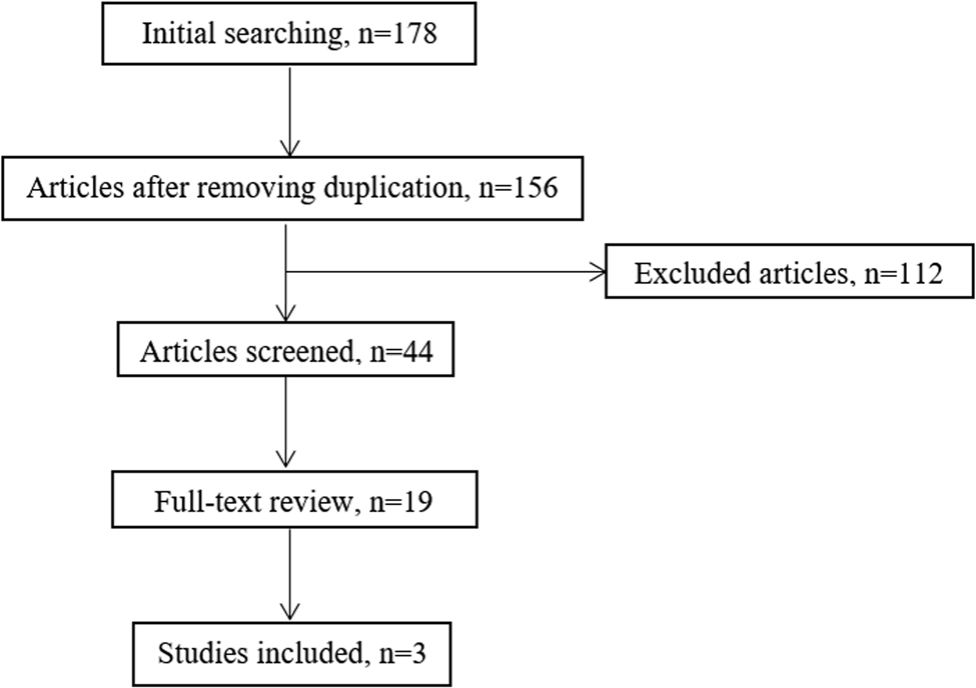
Flowchart of this study.

Based on a search of the mentioned databases, 44 articles were obtained. Next, of 19 published articles, 3 [6,7,8] were eventually included in this article. After reviewing the references of the included studies, no articles were added. A summary of the procedures of the studies selected in this article is detailed in Figure 1.

## 4 Discussion

### 4.1 Iron-overload cardiomyopathy

As iron is crucial for the heart to work incessantly, where iron participates in the mitochondrial energy process of cardiomyocytes, there are multiple ways for iron to be imported into these cells to prevent myocardial iron deficiency. This phenomenon makes the cardiomyocytes prone to iron overload and damaged by the noxious effects of systemic iron redundant [9]. In an iron-overload state, however, circulating hepcidin levels go up to protect the heart against iron accumulation resulting from an increased iron efflux secondary to the upregulation of cardiomyocyte ferroportin (FPN) [10]. Under the iron-overload conditions, the level of labile iron tends to rise, increasing reactive oxygen species (ROS) formation, then making heart and cardiac organelles damaged and dysfunctional. However, in normal-state iron hemostasis, labile iron lingers very low.

In cases with advanced heart failure, mitochondrial iron rises, which brings about oxidative damage in cardiomyocytes [11]. In addition to altered inflammatory mediators, a surged level of deleterious intercellular ROS is thought to be the most key pathogenetic driver determining cardiomyocyte injury through the advancing contingencies of apoptosis, fibrosis, and then systolic and diastolic cardiac dysfunction [10]. In iron-overloaded cases, redundant non-transferrin-bound iron (NTBI) followed by saturated transferrin enters into cardiomyocytes, most probably through calcium channels, which boosts ROS genesis that decreases calcium uptake, then adversely influences cardiac excitation–contraction coupling, causing heart failure and iron-overload cardiomyopathy [10].

Iron overload comes from increased peripheral hemolysis, a hyperabsorption of intestinal iron, ineffective erythropoiesis, and red blood cell transfusion. Transporters playing a significant role in entering iron in cardiomyocytes are the divalent metal transporter 1 (DMT-1), zinc transporters, and T-type and L-type calcium channels (TTCC and LTCC). DMT-1 mediates the mechanism by which iron ions enter enterocytes, and FPN does export iron from the cardiomyocytes, which was also found in the heart [7]. Calcium channels are the primary factor contributing to the development of cardiomyopathy in iron-overload circumstances. Clinical studies have shown that TTCC and LTCC blockers, as typical calcium channel inhibitors, can deter iron influx to cardiomyocytes [6,12]. Amlodipine, for example, may act to cut down the iron uptake from cardiomyocytes in circumstances of iron overload, consequently improving the function of mitochondria. In these patients, it is the overburdened intracellular iron, known as labile iron pool (LIP), that produces enormous ROS [13], leading to the developing disturbed mitochondrial function and then heart failure [14].

### 4.2 Current treatments addressing iron-induced cardiomyopathy

In thalassemia cases, the underlying treatment of cardiac hemosiderosis includes iron-chelating agents, such as deferoxamine, deferasirox, and deferiprone, affecting circulation ferritin levels and iron accumulation in organs through boosting iron excretion. Deferasirox therapy, for example, can improve left ventricular function in thalassemia cases, although co-administration with other iron chelators seems to raise cardiac relaxation time soon in severe cases [15]. Based on existing evidence, deferiprone has more penetrating properties to enter the cardiomyocytes than other agents, leading to a considerable fall in the mortality rate in this population due to more effective cardiac iron sequestration [16,17]. Deferiprone therapy has also displayed advantageous effects in Fe^2+^- and Fe^3+^-overload circumstances [18]. Furthermore, other upsides, such as rapid iron binding, high therapeutic index, and antioxidant effects through mobilizing LIP and constraining iron catalytic activity in ROS development, have provided support for using deferiprone in iron-loading cardiomyopathy [19]. As such, using deferiprone as either monotherapy or combined with other iron chelators has been grossly strengthened [20,21].

As TTCC and LTCC are one of the ways through which iron gets in cardiomyocytes, using calcium channel blockers (CCBs) has shown upsides in falling iron subside in cardiac tissues [22]. Among those agents, amlodipine has depicted a better performance in lessening cardiac hemosiderosis in multiple clinical studies, although the others suffer from a scarcity of research, particularly T-type ones [5]. A recent meta-analysis [23] on seven randomized controlled trials (*n* = 233) has illustrated that myocardial iron concentration can see a dramatic drop following amlodipine therapy (Hedges’ *g*: −0.82, 95% confidence interval [CI]: −1.40 to −0.24), whereas cardiac T2* can ramp up after the amlodipine treatment significantly (Hedges’ *g*: 0.36, 95% CI: 0.10 to 0.62). As one of the adverse effects of using CCBs, the risk of lower limb edema and gastric upset must be paid heed in these patients [23].

### 4.3 Antioxidants and iron-induced cardiomyopathy

Existing evidence has revealed that using antioxidants added to iron chelators can be an effective means of cardiac iron sequestration and cardiac function improvement in iron-overloaded thalassemia patients [24,25,26]. With the synergistic effects of iron chelators in combination with antioxidants for treating iron-associated cardiomyopathy, combination therapy can have a superior performance in these circumstances. To improve cardiac function in iron-overloaded thalassemia cases, antioxidants can be seen as a heat-friendly therapeutic option thanks to replenishing cardiac antioxidants, decreasing iron ingress into the cardiomyocytes, mitigating inflammation, and improving cardiac mitochondrial performance [24,25,26].

Additionally, according to an *in vivo* study, using digoxin, a pharmacological choice for heart failure in thalassemia can cause oxidative stress to elevate as a result of raised iron inflow in cardiomyocytes through calcium ports, especially at higher doses. As such, this question arises whether taking antioxidants, such as vitamin E [27], *N*-acetylcysteine [28], and silymarin [29], alongside standard treatments in thalassemia cases with cardiomyopathy under digoxin therapy, would be beneficial or necessary.

### 4.4 Ebselen

Iron accumulation in cardiac tissue leads to mitochondrial dysfunction and pathological complications, ultimately causing iron-overload cardiomyopathy. Oxidative stress caused by iron accumulation in mitochondria ends up damaging DNA and membrane. Given the role of mitochondria in calcium management and membrane depolarization, oxidative stress in mitochondria causes the disruption of myocardial function and cellular damage, thus resulting in iron-overload cardiomyopathy [30,31,32]. In this condition, the therapeutic approach is the prescription of iron-chelating agents, which focus on cytosolic and tissue iron intake and iron excretion alongside a limited efficacy on mitochondrial iron accumulation, as well as ototoxicity and neurotoxicity on its long-term use.

Ebselen [2-phenyl-1,2-benzisoselenazol-3(2*H*)-one] is an organ-selenium molecule functioning as a particular DMT-1 inhibitor with anti-inflammatory and antioxidant properties [33], a synthetic GPx seleno-nonenzyme, with anti-atherosclerotic and cytoprotective properties against injury [34]. With binding to intracellular iron and reducing ROS synthesis under iron-overload conditions due to antioxidant and anti-inflammatory properties, co-administration of ebselen with iron chelators can have a superiority to ameliorate the mitochondrial iron accumulation that may improve cardiac contractility in this situation [7,35]. Having been called by chemists a pan-assay interference compound, ebselen has to do with its ability to bind non-selectively to reactive cysteines, binding in a nucleophilic manner between aminoacidic thiol and electrophilic selenium. A study showed that the ebselen’s designed probe for cellular targets was suitable for clinical trials. They designed and synthesized biotin–ebselen (biotinylated ebselen probe), and exposure of the probe to HeLa cell lysates showed that biotin–ebselen could bind to 462 proteins [36]. As a novel intervention, ebselen has unveiled benefits in mitigating iron burden in cardiomyocytes, which can improve the cardiac function and decline the mortality rate in iron-overloaded animal models [8,37].

#### 4.4.1 Potential experimental and clinical applications

Kumfu et al., through an *in vivo* study, showed that 1 month co-using ebselen and desferrioxamine could decline cardiac hemosiderosis, cardiac malondialdehyde, and plasma NTBI in thalassemic mice with an iron burden. As a result, an improvement in heart rate variability and left ventricular function was also observed. Likewise, ebselen administered with desferrioxamine decreased liver iron concentration, liver malondialdehyde, plasma malondialdehyde, and mortality rate [37].

A study of mouse models with acute exacerbations of chronic obstructive pulmonary disease was conducted to investigate the role of apocynin and ebselen in lung inflammation. The study results showed that apocynin and ebselen could significantly reduce the expression of protease mRNA, chemokine, pro-inflammatory cytokines, and inflammation in the bronchoalveolar lavage fluid, which represents them in weakening inflammation through targeting oxidative stress [38].

In an ischemic model deprived of oxygen and glucose, including human neuroblastoma and microglia cells, ebselen derivatives were evaluated for their neuroprotective, antioxidant, and anti-neuroinflammatory effects [39]. At similar concentrations, ebselen derivatives exposed to microglial cells reduced the production of nitric oxide and tumor necrosis factor-alpha. Some ebselen derivatives exposed to human neuroblastoma SH-SY5Y cells could increase cell survival rates by 84% [39]. Moreover, a study investigating the inhibitory function of ebselen and its derivatives on SARS-CoV-2 proteases, including main protease (M^pro^) and papain-like protease (PL^pro^), determined that ebselen and its derivatives could inhibit both proteases PL^pro^ and M^pro^ [40]. High cytoprotective activity and the highest antiviral property have been reported with ebselen diselenide derivatives. In another piece of their work, they displayed that 11 ebselen analogs have an antiviral cytoprotective activity with low cytotoxicity [40].

In a study, the effect of ebselen on amyloid precursor protein (APP) processing in human neuroblastoma SH-SY5Y cells was also evaluated [41]. Considering that iron has an increasing impact on amyloidogenic metabolism, this study was performed to explore the inhibitory role of ebselen as a DMT-1 inhibitor in regulating APP processing. The results suggested that ebselen can lessen iron uptake and subsequently decline ROS production, suppressing β-amyloid generation. They argued that ebselen might be an appropriate option for Alzheimer’s disease, both prophylactically and therapeutically [41].

In a crossover trial (*n* = 26) [42], ebselen, due to its GPx-mimicking role, was conducted in diabetic patients – type 1 and type 2 – aiming to evaluate its effects on oxidative stress and vasodilation. The oral form of ebselen was administered at a dose of 150 mg every 12 h for 4 weeks, with a washout period in between (4 weeks). The results displayed no significant changes in oxidative stress levels and vasodilation following treatment with ebselen [42].

According to the history of ebselen’s effectiveness in reducing temporary and permanent noise-induced hearing loss in preclinical findings, oral formulations of ebselen in doses of 200 mg (*n* = 22), 400 mg (*n* = 20), and 600 mg (*n* = 21) and placebo (*n* = 20) were prescribed every 12 h for 4 days, through research conducted in young adults [43]. The effectiveness of ebselen in preventing temporary threshold shift (TTS) induced by noise was observed only at a dose of 400 mg. At 4 kHz, the mean TTS in the 400 mg ebselen-treated group showed a 68% reduction compared with the placebo group, 1.32 dB (SE 0.91) versus 4.07 dB (SE 0.90) (difference: −2.75 dB, 95% CI: −4.54 to −0.97) [43].

Based on safety, well tolerability, and anti-inflammatory characteristics of ebselen, a study was performed to probe its efficacy as an alternative to lithium in treating manic or hypomanic patients due to lithium toxicity and poor tolerability at a dose of 600 mg every 12 h for 3 weeks [44]. Although the Young Mania Rating Scale and Altman Self-Rating Mania Scale observed improvements after the administration of ebselen, the betterments were not statistically significant. In contrast, the improvement experienced in the Clinical Global Impression-Severity Scale after 3 weeks of the treatment was statistically significant (adjusted mean difference: −0.58, 95% CI: −1.14 to − 0.03) [44].

In an experimental study, Davis et al. demonstrated that ebselen can imitate glutathione GPx properties protecting cardiac function and increasing survival [8]. They divided 15 mice into three groups: those who received intraperitoneally normal saline as controls (0.1 mL normal saline i.p. per mouse, per day), the iron-only regimen group (10 mg iron dextran i.p. per mouse, per day), and iron plus ebselen treatment (25 mg/kg p.o. per mouse, per day) which the injection duration of the doses was done 7 days a week for 20 days. The ROS and iron absorption levels in cardiomyocytes were lowered in iron-overloaded animals treated with the ebselen, while GPx activity escalated. The animals treated with ebselen experienced a 19% drop in iron concentration and a 48% rise in GPx activity compared to the iron-only regimen group.

#### 4.4.2 Action mechanisms of ebselen

The effectiveness of ebselen in improving parameters regarding iron-induced cardiotoxicity has been demonstrated on account of being a DMT-1 inhibitor and antioxidant, thanks to a reduction in ROS production in cardiomyocytes under excessive iron accumulation [7]. An *in vivo* study with ebselen discovered that it effectively reduced myocardial infarct size by affecting the process of ischemia–reperfusion injury. They pointed out the waned apoptosis and declined oxidative stress as its functional mechanism. Most probably, the antioxidative property of ebselen is mediated through the nitric oxide pathway associated with an increase in the activity of superoxide dismutase (SOD) and GPx, as well as a reduction in the protein carbonyls and malondialdehyde concentrations. To be more precise, ebselen therapy may decrease the expression of pro-apoptotic proteins, elevate the expression of anti-apoptotic proteins, and inhibit the expression of C-Caspase-3, C-Caspase-8, C-PARP, and Bax proteins, alongside an increase in the expression of Bcl-2 protein. In addition, taking ebselen has been reported to be accompanied by a decrease in the expression of pro-apoptotic proteins and an increase in the expression of anti-apoptotic proteins [33,45].

Superfluous free radicals ensuing from iron surplus play an important role in lipid peroxidation, usually concomitant with reduced levels of GPx, SOD, and catalase enzymes [46]. As a consequence, myocardial damage is regarded with a decreased activity of the GPx and SOD enzymes [33]. Direct ROS-mediated injury and the cardiac mitochondrial dysfunction caused by oxidative stress are also responsible for cardiotoxicity in iron-overloaded cases [17,47]. Ebselen may reduce H_2_O_2_ levels and inhibit Fe^2+^ and H_2_O_2_ reaction, which subsequently mitigates the genesis of hydroxyl radicals, then reduces lipid peroxidation and aldehyde production. Besides, GPx, a selenium-dependent enzyme and one of the main antioxidants in the heart, scavenges peroxidases and H_2_O_2_ that prevent the formation of free radicals in the Fenton reaction. As such, evaluating cardiac GPx activity has determined that ebselen can increase antioxidant stores and avoid cardiomyocyte damage under enhanced oxidative stress secondary to iron overload [8].

An *in vivo* study showed that ebselen was found to impede lipid peroxidation and iron deposition and scale up impaired ferroptosis and programmed cell death via inhibiting the activity of DMT-1. Ebselen inhibits DMT-1 transporters, obstructing iron influx into cardiomyocytes, then leading to decreasing intracellular oxidative stress. It has been shown that ebselen can lessen hepcidin expression acting as an inducer of ferroptosis in iron metabolism. As ebselen causes hepcidin to decrease, the expression of FPN1, an intracellular iron exporter, tends to rise, and the activation of DMT-1 signaling will be declined, resulting in diminishing LIP in cardiomyocytes [48]. Furthermore, decreasing malondialdehyde levels and increasing glutathione and GPx4 activity are mediated by ebselen, affecting intracellular oxidative stress balance, and, subsequently, ferroptosis will be undermined [48].

#### 4.4.3 Side effects of ebselen

Ebselen is being investigated in clinical trials as a potential therapy for stroke, hearing loss, and bipolar disorder with good safety profiles and no adverse effects [43,44,49]. In a trial implemented on diabetic cases, ebselen was well tolerated and illustrated a safe behavior with no serious adverse effects, only one case of an allergic reaction (mild form) [42]. Moreover, over previous phases I [50] and II [43] of field trials conducted on healthy adults aged 18–31, taking ebselen at 200, 400, and 600 mg twice daily was safe and well tolerated. Besides, the prescription of ebselen at 600 mg twice daily for 3 weeks in manic or hypomanic cases did not bring adverse effects or harmful complications to the study subjects [44].

However, according to two clinical trials, the prevalence of drowsiness among ebselen users was reported between 14 and 25% [44,51]. Using ebselen 600 mg twice daily (*n* = 33) in mania or hypomania cases brought 6% pruritus, 13% upper respiratory tract infection, 2% abdominal pain, 8% insomnia, 5% rash, and 4% frequent urination more than in the placebo group [44]. Monitoring these potential side effects is therefore highly invigorated for the subsequent studies.

### 4.5 Future directions

Future clinical trial studies are required to evaluate the safety and efficacy of ebselen in thalassemia patients with cardiac complications with a large sample size so that its clinical significance can be discussed more precisely. To elucidate the cardioprotective effects of ebselen evidently, more weight requires to be given to the dose and duration of ebselen administration as monotherapy or in combination, for instance, with CCBs. The relationship between ebselen doses and patients’ responses is of particular importance and needs to be gauged and reported in the future studies. In addition, determining drug levels in patients treated with ebselen will provide insights into the concurrent prescribing of treatment regimens, demonstrating the interaction of treatment regimens, including the effect of ebselen on plasma levels of other drugs, and *vice versa*. The incoming research should consider other factors, such as inflammation or genetic mutations, influencing disease progression significantly.

As cardiomyocytes are poorly protected from iron surplus in these cases, adopting a new clinical approach using ebselen in conjunction with routine treatment may slash the risk of iron-overload-induced cardiac dysfunction, which can hopefully raise the patients’ lifespan. Future work can focus on investigating how ebselen affects mitochondrial function and calcium homeostasis within the heart muscle cells under conditions of excess iron accumulation. Therefore, designing and conducting more well-designed studies determining the clinical impacts and the adverse effects of ebselen are required to be encouraged. In closing, we can urge pharmacologists to make a significant contribution in preparing this medication on a large scale to be widely available to therapists; maybe afterward, its merit will be proved by passing phase I and II clinical trials that are imperative prior to Food and Drug Administration approval in this context.

## 5 Conclusion

Intriguingly, the properties of ebselen, such as reducing iron entry by inhibiting DMT-1 and increasing FPN1 expression, and antioxidant characteristics by mimicking the function of GPx, make it an exciting and promising possibility for adjuvant therapy in patients with thalassemia alongside the standard treatment with iron chelators, particularly in severe cases with cardiomyopathy. However, clinical trials recruiting human subjects need to be carried out ahead of definitive conclusions depicting its effectiveness and safety.
